# The small-world network of global protests

**DOI:** 10.1038/s41598-021-98628-y

**Published:** 2021-09-28

**Authors:** Leonardo N. Ferreira, Inho Hong, Alex Rutherford, Manuel Cebrian

**Affiliations:** Center for Humans and Machines, Max Planck Institute for Human Development, Lentzeallee 94, 14195 Berlin, Germany

**Keywords:** Complex networks, Computational science

## Abstract

Protest diffusion is a cascade process that can spread over different regions of the planet. The way and the extension that this phenomenon can occur is still not properly understood. Here, we empirically investigate this question using protest data from GDELT and ICEWS, two of the most extensive and longest-running data sets freely available. We divide the globe into grid cells and construct a temporal network for each data set where nodes represent cells and links are established between nodes if their protest events co-occur. We show that the temporal networks are small-world, indicating that the cells are directly linked or separated by a few steps on average. Furthermore, the average path lengths are decreasing through the years, which suggests that the world is becoming “smaller”. The persistent temporal hubs present in both data sets indicate that protests can spread faster through the hubs. This topological feature is consistent with the hypothesis that protests can quickly diffuse from one region to any other part of the globe.

## Introduction

Protests are a powerful tool of transformation that has been shaping societies^1,2^. Typical forms of protests include strikes, boycotts, passage obstructions, and violent acts for different reasons such as leadership change, policy adjustments, institutional and regime alterations^3–5^. Some protests are small and localized, while others quickly spread to other places and can achieve regional or global proportions. High-profile examples include the Black Lives Matter movement or the Arab Spring^6–8^. Traditional media always had a fundamental role in this spreading process, but the advent of social media changed the way information spreads, also impacting protests diffusion^9–13^. The emergence of online social media platforms and their easy access via mobile devices permitted not just fast communication but also quick mobilization. These platforms have an important role in direct protests organizations but also have introduced or amplified some features and behaviors like bots, recommendation algorithms, fake news, internet trolls, and echo chambers that can also influence users to engage in protests^14–16^. The way and extension of how protests diffuse in this complex scenario of fast information spreading are still not well understood.

Network science has been successfully applied to study complex systems in different research areas. One of the most interesting features observed in many of these networks is the small-world effect^17–19^. This phenomenon was initially investigated in sociology but was also observed in many different domains. It comprehends the idea that people around the world are on average connected by a few acquaintances, the so-called six degrees of separation^20^. This number has been studied by many previous works, which indicate that the world is becoming “smaller”, mainly caused by online social networks^21–23^. These results reconfirm that the internet can quickly spread information around the globe, but innumerable consequences of this feature are still unknown.

In this paper, we use data from GDELT^24,25^ and ICEWS^26^, two of the most extensive and longest-running data sets of protest events, to measure how protests are linked between different regions in the globe. We are not aware of previous works that have analyzed protests using such a large volume of data. We construct a temporal functional network of protests for each data set by dividing the globe into grid cells, represented by nodes, and links are established between cells with co-occurrent protests^27,28^. The main advantage of this approach is the possibility to analyze protests and their dynamic relationship inside and between countries. Using statistical network measures, we describe these two worldwide networks of protests, which reveal a small-world effect. It means that any pair of cells are directly linked or are separated by a few links on average. This result suggests that a protest event in any part of the globe can be quickly disseminated to any other part of the world. We present in the rest of this paper our results in detail.

## Results

**Figure 1:**
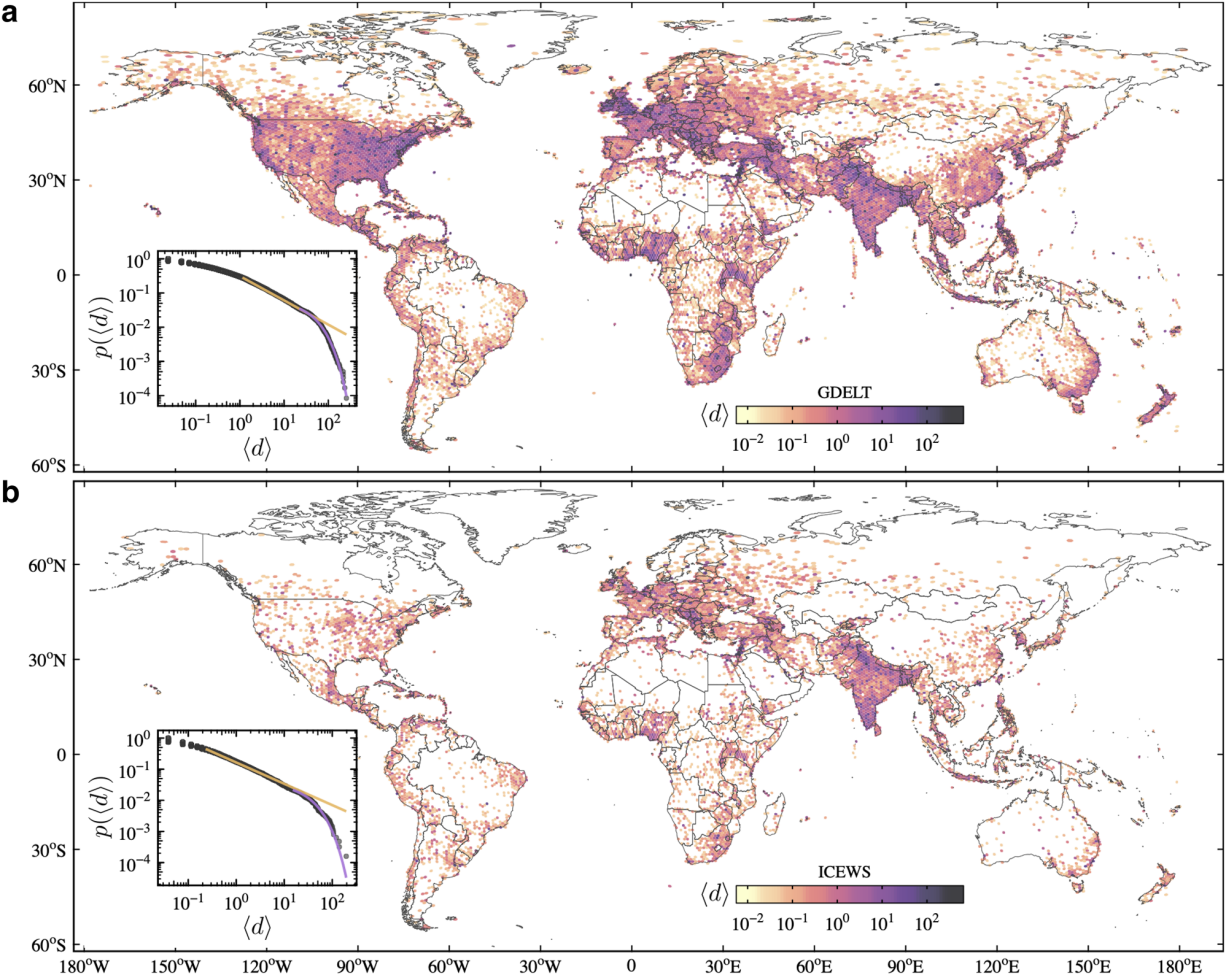
The average number of days with protests by year ($\langle d \rangle$) for each cell in the (**a**) GDELT (1979–2020) and (**b**) ICEWS (1995–2020) data sets. The color scale is logarithmic. The inner plots show the probability distribution of $\langle d \rangle$ also in a logarithmic scale. The orange and purple lines correspond to power-law and exponential fittings, respectively^34^. Maps were generated using R (version 3.6.2)^44^ and the ggplot2 (version 3.3.2)^45^ package.

First, we present an overview of both data sets used in our experiments. Figure 1 shows the average number of days with protest events by year ($\langle d \rangle$) for each cell. One major difference is the number of cells with events in both data sets. ICEWS has 6266 cells, while GDELT has 11912. Some previous works have already presented this difference which is mainly caused by the way they are curated^25,29^. GDELT and ICEWS are constructed by automatically processing news media to extract and identify events using different algorithms. While GDELT has some duplicate events, ICEWS misses some events. Therefore, an intermediary scenario between both data sets should be more realistic. This known difference is the motivation to use two data sets and to consider both ones when drawing conclusions. Besides their differences, both data sets seem to capture the main protest events. Figure 1 also shows some similarities in the spatial distribution of $\langle d \rangle$. Both GDELT and ICEWS capture a high average number of days with protests in India, Nigeria, South Africa, Israel, and many European countries. Conversely, GDELT captures many more days with events in the US than ICEWS. One possible explanation for this difference is that ICEWS does not share all data, but only a part of the events that has been sanctioned by the US Government for public release^30^. The correlation between $\langle d \rangle$ in both data sets considering all the cells with events in both data set is 0.68, which also indicates similarities.

The two probability distributions of $\langle d \rangle$ (Fig. 1 inner plots) can be partially approximated by power-laws with exponential cut-off indicating that the vast majority of cells have only a few days with events. It means that 95% of the cells in GDELT and ICEWS have less than 14 and 5 days with protests in the year on average, respectively. In those days with protests, the larger fraction comprises single events while a minority has spikes. A very few cells have a high number of days with events. Many of those cells account for metropolitan areas and highly populated regions around the world. Other cells, in a much lower number, represent incorrect entries in the data set, especially in GDELT. One example of incorrect events occurs in the cell in origin ($0^{\circ }$, $0^{\circ }$) that is caused by the missing event location. Another similar issue occurs when only the event country is known, but not the precise location. These events receive the geographic centers of the respective countries.

**Figure 2:**
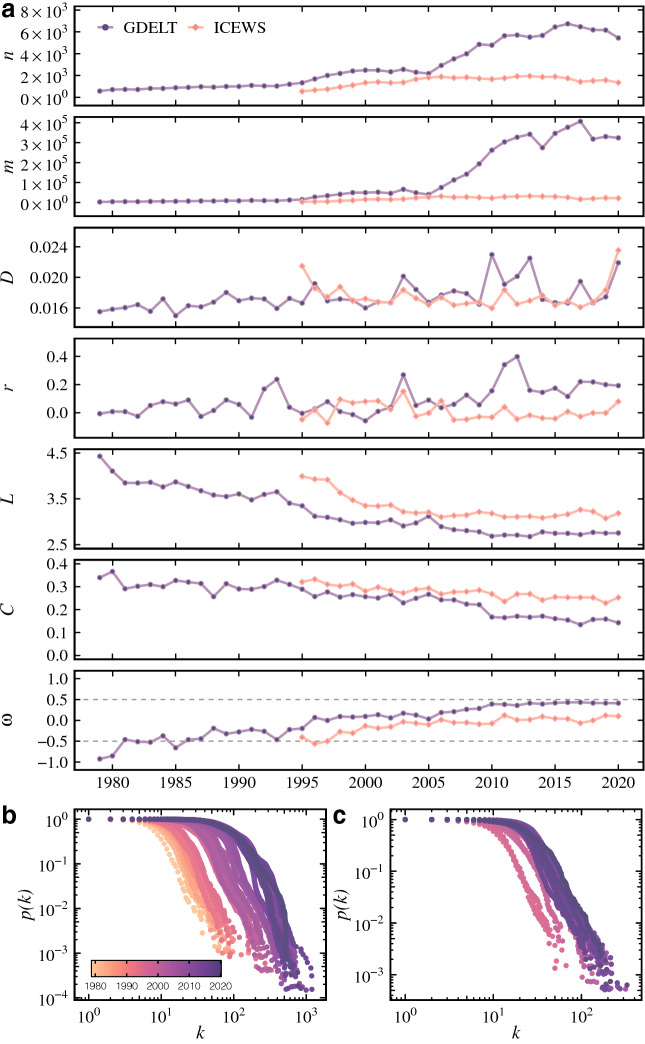
Temporal networks characterization using (**a**) global statistical measures as the number of nodes *n*, links *m*, edge density *D*, assortativity *r*, average path length *L*, clustering coefficient *C*, small-worldness $\omega$, and the degree distributions for the (**b**) GDELT and (**c**) ICEWS data sets. The size of the largest components in both temporal networks corresponds to at least 96% of the number of nodes (cells). Thus, for simplicity reasons and without loss of much information, we report the measures for the largest component in each snapshot.

We construct a temporal network for each data set where nodes represent cells and links connect cells with co-occurrence of protests. Figure 2 describes these networks using global statistical measures applied to the largest component of each snapshot^31^. We consider the largest components for simplicity reasons and without considerable loss of information since they correspond to at least 96% (minimum value observed) of the number of nodes in the respective snapshot. The number of nodes *n* represents the count of cells in each year. All the cells in each snapshot are linked with at least another one since we consider only each snapshot’s largest component.

From 1979 to 1996, the number of cells for GDELT is around 500 and remains stable until 1996 where *n* starts to increase. The years of 2005 and 2006 mark a considerable increase in the number of nodes and links in the GDELT network. This period corresponds to the emergence of social media platforms like YouTube, Facebook, and Twitter and a huge increase in mobile internet access^32^. These platforms completely changed the way humans communicate and permitted an easy and quick spread of information and social mobilization^33^. Evidence also indicates that online social networks played an important role in protests organizations^9,10,12^. The rise of social platforms is one probable explanation for the increasing number of nodes, links, and connected countries. Different from GDELT, ICEWS has remained practically stable since the beginning (1995). These divergent results are directly related to the difference in the number of events captured by both data sets (Fig. 1). As previously mentioned, former studies also observed this difference and we consider it a strong reason to analyze them concomitantly.

The edge density (*D*) in all the snapshots remains between 1.4% and 2.3%, which shows that both networks are sparse. The degree assortativity (*r*) is close to zero in most parts of the historical period, indicating no strong tendency for nodes to connect to similar ones. GDELT shows an increasing tendency in *r* after 2005, showing that the new nodes and links observed in the same period have a weak tendency of connecting to other nodes with the same degree. The assortativity values also denote that the difference in the number of days with events (Fig. 1) does not have a notable impact on the connectivity. Thus, cells with more days with events do not have a higher probability of connecting to cells with fewer events in general.

From the evolution of the networks, it is possible to observe that the average path length (*L*) is small and decreases with the years. It means that any pairs of cells are directly connected or are separated by a few steps. The global clustering coefficient (*C*) also decreases during the years. Despite the decrease, all *C* values can be considered high compared to their respective randomized or lattice-like versions. It indicates that the networks present a considerably high number of triangles (3-vertex cliques). The small average path length and the high clustering coefficient indicate that the temporal network is small-world^17^. To investigate this hypothesis, we also present in Fig. 2 the small-word index $\omega$^18^. The idea behind this index is to compare the observed networks to the original description of small-world networks as defined by Watts and Strogatz^17^. According to the definition of $\omega$, negative values denote that the networks are more regular (lattice-like) while positive ones indicate networks with more random characteristics. Values close to zero, i.e., $\omega \in [-0.5,0.5]$, mean that the network is small-world. From the evolution of $\omega$, we observe that both networks are in the interval of small-world networks. They start with $\omega$ lower or close to $-0.5$ and increase to 0.5 in GDELT and zero in ICEWS. This difference between data sets indicates that the GDELT small-world network has more characteristics of a random topology. In contrast, ICEWS has the expected high clustering and low path length as in a small-world network.

When we analyze the degree distributions of each snapshot (Fig. 2b), we can discard the hypothesis of Poisson degree distributions ($p\,\hbox {value} < 0.01$)^34^. These distributions indicate that the topology does not follow the Erdős-Rényi random graph model^35^. Other distributions can be considered instead. Power-law, log-normal, and exponential distributions are more plausible ($p\,\hbox {value} > 0.1$) depending on the year. These distributions are characterized by many low-degree nodes but just a few highly connected nodes (hubs). In some years where a power-law distribution ($p(k) \approx k^{-\gamma }$) is a plausible assumption, the degree exponents $\gamma$ stay in the random regime ($\gamma > 3$)^19^. This is indicative that the number of hubs is smaller than expected in a scale-free network. Therefore, we cannot claim that the network has a scale-free nature ($2< \gamma < 3$). However, in the three distributions, the hubs decrease the distance between the nodes (small-world) but do not drastically as in a scale-free network (ultra-small)^36^. The same stands for the log-normal and the exponential distributions. The observed degree distributions are another indicator of a small-world effect in the network.

**Figure 3:**
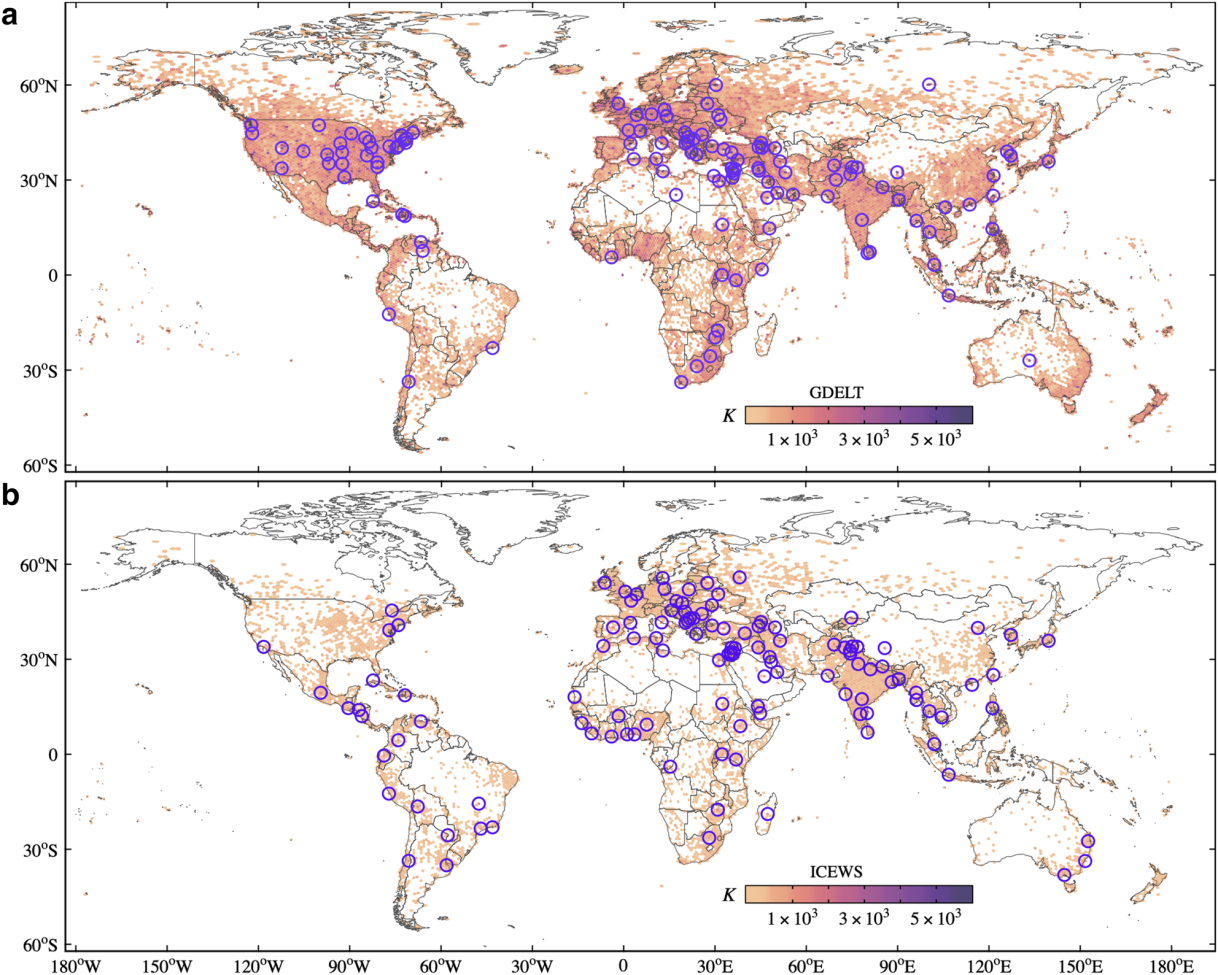
Hubs in the temporal networks. Circles highlight the 120 hubs that correspond to the 99th and 98th percentiles of the highest temporal degrees (*K*) for GDELT and ICEWS, respectively. Maps were generated using R (version 3.6.2)^44^ and the ggplot2 (version 3.3.2)^45^ package.

Hubs play an important role in the co-occurrence of protests between cells because they decrease the average distance between nodes. We have shown that both temporal networks have high degree nodes in all layers. Next, we investigate the locations of the hubs. Fig. 3 presents the aggregated degree *K* (sum of temporal degrees), which can be used to find persistent hubs (high *K*). The temporal degree of all nodes is illustrated in Supplementary Fig. S1 and can be found online. Hubs mainly correspond to cells with high population densities or important local centers as state and national capitals. The correlation between the aggregated degree in both data sets is 0.68, which indicates similarities. To illustrate the similarity, we highlight the 120 nodes with the highest aggregated degree in both data sets in Fig. 3. From these 120 hubs, 56 appear in both data sets. The differences are intrinsically related to the number of events captured by both data sets. GDELT has many more hubs in the US than ICEWS, while ICEWS shows more hubs in India than GDELT. ICEWS also has the hubs more spread worldwide, exhibiting more hubs in South America, Central America, and Africa.

## Discussion

In this paper, we used data from two of the most extensive event data sets available to evaluate the co-occurrence of protests in different regions of the planet. Our goal was to measure event co-occurrences between regions and estimate how protest diffusion may occur worldwide. As far as we know, no previous work has analyzed protests using such a large amount of data. Our approach consisted of mapping the data from ICEWS and GDELT into respective temporal networks and measuring their features to describe protest co-occurrences around the globe. It is important to state that co-occurrence does not imply causality. All results and conclusions here presented rely exclusively on co-occurrences, which cannot be interpreted as causal relationships of protests between cells.

Our approach permitted the comparison between both data sets and to detect differences and similarities. One of the evident distinctions is the considerable increase in the number of events in GDELT after 2005 that ICEWS does not capture. This difference makes it evident that there are limitations in the construction of these data sets. Previous studies also observed this difference and suggested that an intermediary scenario between GDELT and ICEWS is more realistic^25,29^. Our results are aligned with these results and reinforce the idea that both data sets should be taken into account when analyzing protests. These differences also motivate future improvements in GDELT and ICEWS, and the development of new open-access data sets.

One interesting similarity between the data sets is the small-world phenomenon that comprehends the small average path length and high clustering coefficient in the temporal networks. The small average path length is mainly caused by the temporal hubs that connect many cells. The presented results do not explain the multiple specific reasons that make a particular hub connect to many other cells. Our results also do not present explanations about why a cell becomes a hub. We believe that multiple particular factors lead one cell to connect to many other ones and become a hub in the context of protests. Our goal is not to describe these many particular reasons. Furthermore, these factors are probably not recent and occurred in the long historical development of the major cities. GDELT and ICEWS, two of the largest protest data sets, started in 1979 and 1995, respectively. These years are very recent compared to the age of most cities in the world. Many cities have been founded centuries ago and have passed through many transformations. For this reason, we believe that it is improbable that any cell would become a hub in this small period of time. This can be seen by the persistence of the temporal hubs since the beginning of the data sets. Hubs probably became hubs before the period covered by the data sets.

We cannot explain the reasons why cells become hubs, but we can identify them. Using our approach, we can detect worldwide hubs and study protest diffusion patterns from a macroscopic point of view. As a general conclusion, our results indicate that temporal hubs decrease the average distance between nodes. Considering three event times series $X_1$, $X_2$ and $X_3$, if $X_1$ has co-occurrent events with $X_2$ and $X_2$ has co-occurrent events with $X_3$, there is a high probability of $X_1$ and $X_3$ will also have co-occurrent events. This transitivity effect is observed in the considerably high clustering coefficient in the networks. This is indicative that protests can start in any region (leaf nodes or hubs) and quickly spread (through the hubs) to any other cell. It is important to reaffirm that co-occurrences do not imply causal effects, which cannot be interpreted from our results. We conclude that the small-world effect observed in the temporal network is consistent with the hypothesis that protests can quickly diffuse from one region to any other part of the globe through the hubs. Forecasting methods in future works can incorporate this result in order to make better predictions.

## Methods

### Data

We used data from the Global Database of Events, Language, and Tone (GDELT)^24^ and the Integrated Crisis Early Warning System (ICEWS)^26,^ which arguably are the largest open repositories of geolocated events. The data sets are constructed by processing worldwide news media in different formats such as print, broadcast, and online in over 100 languages. Events are extracted from media and are categorized following the Conflict and Mediation Event Observations (CAMEO) framework^37^. In our experiments, we considered only protest events (CAMEO code 14), which include demonstrations, strikes, boycotts, obstructions, and violent protests for different reasons like leadership change, policy change, institutional and regime changes, and other non-categorized protests. We used all the available data from both data sets, which comprehends data from 1979-2020 in GDELT and 1995-2020 in ICEWS.

Events in origin cell ($0^{\circ }$, $0^{\circ }$) can be caused by missing locations about the protests, while those ones located in the geographic centers of countries have just information about the country but not the precise location. In our analysis, we opt to disregard only the cell at the origin.

### Network construction

We used protest data from GDELT and ICEWS to construct two temporal networks where a network snapshot represents each year from the historical period. For each year, we divided the globe into hexagonal grid cells^38^. We opted for a hexagonal grid instead of a rectangular longitude-latitude grid because it generates a more uniform coverage and avoids distortions. The hexagonal grid system has some possible resolutions that define the number of cells and the cell area. In our experiments, we opt for the resolution eight that generates 65,612 cells of approximately 7,774 $km^2$ each. The main reason for our choice is that the cell area covers the most major urban areas^39^.

Only those cells with at least one event in the year were considered. For each cell, we extracted an event time series $X = \{x_1,\dots ,x_n\}, x_i \le x_{i+1}$ where $x_i \in [1,365]$ represents a day with events in the year. The event co-occurrence between cells was measured using the van Rossum distance with a Laplacian smoother^40–42^. Every event times series *X* is mapped to a function $f(t;X) = 1/n\sum _{i=1}^{n}h_{\tau }(t-x_i)u(t-x_i)$, where $h_{\tau }(t) = \exp (-|t|/\tau )/(2\tau )$ is the Laplacian kernel smoother and *u*(*t*) is the Heaviside step function. The parameter $\tau$ defines the co-occurrence time scale. In our experiments, we carefully choose $\tau = 7$ because it generates network layers that keep only significant links and do not break them into smaller components. Furthermore, we assume that one week is a plausible maximum delay between two co-occurrent protest events. In the last week of the year, $\tau$ decreases one unit until the last day of the year where $\tau = 0$. It means that co-occurrences in the last week of one year and the first week of the next year are not considered. This choice simplifies the construction and does not have much impact on the results.

Given two event time series *X* and *Y*, their distance is defined as the Euclidean distance of their mapping function, e.g., $d(X,Y)^2 = \int _{0}^{\infty } [f(t;X) - f(t;Y)]^2\hbox { d }t$. For each pair of event time series, we estimate the statistical significance of event co-occurrence by uniformly randomizing the days with events in both time series and calculating the distance between the surrogate time series. We repeated this process 1000 times to construct a null-model distribution. The events in two cells are considered statistically co-occurrent if their Rossum distance is lower than the 1st percentile. Each snapshot of the temporal network is constructed using nodes to represent cells, and a link is established between two nodes if the respective event time series in the specific year are statistically co-occurrent, considering a significance level of 0.01.

The network construction process requires the calculation of the van Rossum distance to all combinations of time series. For each pair of time series, we calculate 1000 additional distances using the shuffled time series to estimate the link significance. Therefore, the time complexity is combinatorial, which makes this approach computationally very expensive. For example, the construction of GDELT and ICEWS temporal networks require approximately 2.6 $\times$ $10^{11}$ and 3.1 $\times$ $10^{10}$ distance calculations respectively. The large amount of data, high grid resolution, and combinatorial nature of the problem turn infeasible many analyses that are trivial for smaller data sets. An alternative would be to sample the cells and construct smaller versions of the network, but this procedure requires repetitions, which is also unfeasible. Given high time complexity, we opt for selecting the network construction parameters carefully and not present a sensitivity analysis.

We estimate the link significance for every pair of time series in the network construction process. The high number of significance tests increases the probability of spurious links in the network. One traditional approach to dealing with this problem involves *p* values adjustment using techniques such as Bonferroni’s or Šidák’s methods. These methods typically lower the significance threshold, which can be problematic when the number of comparisons is enormous, as in our case. A small significance threshold decreases the number of spurious links but also removes real ones. On the other hand, higher thresholds keep the real links but also the spurious ones. Some approaches were proposed to deal with large-scale multiple tests, but they generally do not entirely remove spurious links. For this reason, we opt to assess evidence that the alternative hypotheses are true. We use a test to compare the observed number of links with the expected number of spurious links, considering the significance threshold of 0.01. The detailed explanation and results can be found online in Supplementary Information (Tab. S1). In summary, the tests show that it is highly probable that some of the significant links in all layers from both data sets are real (alternative hypothesis holds). Even if the expected spurious links occur, it would be improbable that all of them would occur in the hubs. Therefore, it is not expected that spurious links would change the results and conclusions.

### Network analysis

The snapshots are formed by a single component in most of the years. In some years, the snapshots are disconnected, but the size of the largest component in each of these snapshots still corresponds to at least 96% of the respective total number of nodes. Therefore, we opt to consider the largest components in these cases since they still reflect the vast majority of cells with protests each year.

For each network snapshot, we calculated the small-world index $\omega = L_{\text {rand}}/L - C/C_{\text {latt}}$^18^ where *L* is the average path length of the snapshot and $L_{\text {rand}}$ is the median of the average path length values obtained by 200 randomized versions of the snapshot. *C* is the clustering coefficient of the snapshot and $C_{\text {latt}}$ is the maximum clustering coefficient obtained by 200 “latticized” versions of the snapshot. Values of $\omega = [-0.5,0.5]$ indicate a small-world network.

The temporal degree $k_i^{t}$ of node *i* is the number of links it has in snapshot *t*. The aggregated degree $K_i$ of node *i* corresponds to the sum of its temporal degrees considering all snapshots, i.e., $K_i = \sum _{t=1}^{T}k_i^{t}$, where *T* is the total number of snapshots. Hubs in the temporal network are those nodes with the highest aggregated degrees.

## Supplementary Information

Supplementary Information.

## Data Availability

Both data sets (GDELT and ICEWS) used in this paper are freely available for download^30,43^.
