# Supplementary material for: The small-world network of global protests
Source: Sci Rep. 2021 Sep 28;11:19215. doi: 10.1038/s41598-021-98628-y (PMC8479126; doi:10.1038/s41598-021-98628-y)
Supplement: Supplementary file 1 — Supplementary Information. [file 41598_2021_98628_MOESM1_ESM.pdf]

Supplementary Information

The small-world network of global protests

Leonardo N. Ferreira*, Inho Hong, Alex Rutherford, Manuel Cebrian

Center for Humans and Machines, Max Planck Institute for Human Development,
Lentzeallee 94, Berlin 14195, Germany
*ferreira@mpib-berlin.mpg.de

Assessing spurious links

We apply a meta-test that evaluates the probability of the observed number of significant tests (network links) compared to the expected number of false positives (spurious links) if all the time series were independent. If the tests are independent, we assume that the probability of observing a number of significant results ($\alpha = 0.01$) when all null hypotheses are true follows a Poisson probability distribution. If the number of observed significant links is larger than expected according to the probability distribution, then it is highly probable that some of them are real links (alternative hypothesis holds). For example, for GDELT in 2020, there are 5,442 times series. Considering that the 5,442 time series are independents, the number of combinations is 14,804,961 and it is expected 148,049 (1%) spurious links. The observed number of significant links is 324,270. Considering a Poisson probability distribution with mean 148,049, the probability of finding 324,270 spurious links is nearly zero. Therefore, it is highly probable that some of the links are real (alternative hypothesis holds). We repeated this process to all years (layers) in both data sets, and we present the results in Tab. S1. According to this analysis, it is highly probable that some of the significant links in all layers from both data sets are real. We would like to remember that the expected number of spurious links (Tab. S1) does not necessarily imply that this is the fraction of spurious links in the observed links. We also would like to remember that the small-world effect observed in our results is mainly caused by hubs that have many more links than the other cells. Even if the expected spurious links occur, it would be improbable that all of them would occur in the hubs and would not change the results and conclusions.

Table S1: Probability of observed significant links.

Year	GDELT				ICEWS			
	Number of time series	Observed Links	Expected spurious Links	Prob. Observed Sig. Links	Number of time series	Observed Links	Expected spurious Links	Prob. Observed Sig. Links
1979	584	2,554	1,702	0				
1980	708	3,950	2,502	0				
1981	729	4,244	2,653	0				
1982	719	4,192	2,581	0				
1983	815	5,161	3,317	0				
1984	813	5,640	3,300	0				
1985	883	5,823	3,894	0				
1986	911	6,737	4,145	0				
1987	972	7,601	4,719	0				
1988	932	7,269	4,338	0				
1989	996	8,892	4,955	0				
1990	997	8,395	4,965	0				
1991	1,090	10,235	5,935	0				
1992	1,045	9,332	5,454	0				
1993	1,035	8,509	5,350	0				
1994	1,199	12,360	7,182	0				
1995	1,332	14,700	8,864	0	567	3,160	1,604	0
1996	1,685	27,200	14,187	0	665	4,090	2,207	0
1997	2,004	33,961	20,070	0	746	4,829	2,778	0
1998	2,188	41,056	23,925	0	945	8,314	4,460	0
1999	2,409	49,267	29,004	0	1,114	10,426	6,199	0
2000	2,486	49,390	30,888	0	1,337	15,336	8,931	0
2001	2,479	51,538	30,714	0	1,402	16,429	9,821	0
2002	2,344	45,820	27,459	0	1,334	14,840	8,891	0
2003	2,559	65,906	32,729	0	1,361	16,989	9,254	0
2004	2,301	48,680	26,461	0	1,656	23,656	13,703	0
2005	2,181	39,783	23,772	0	1,807	26,771	16,317	0
2006	2,919	75,303	42,588	0	1,887	30,917	17,794	0
2007	3,522	112,984	62,004	0	1,781	25,946	15,850	0
2008	3,987	142,073	79,460	0	1,828	27,683	16,698	0
2009	4,849	193,820	117,539	0	1,724	24,863	14,852	0
2010	4,779	262,237	114,170	0	1,665	22,117	13,852	0
2011	5,639	303,596	158,963	0	1,758	28,357	15,444	0
2012	5,704	327,367	162,649	0	1,923	30,474	18,480	0
2013	5,516	342,271	152,103	0	1,949	32,093	18,983	0
2014	5,669	274,870	160,659	0	1,866	30,662	17,400	0
2015	6,444	346,628	207,593	0	1,900	29,446	18,040	0
2016	6,736	377,437	226,834	0	1,739	25,507	15,111	0
2017	6,467	406,887	209,078	0	1,428	16,398	10,188	0
2018	6,180	318,233	190,931	0	1,517	19,294	11,498	0
2019	6,158	330,651	189,574	0	1,578	22,829	12,442	0
2020	5,442	324,270	148,049	0	1,344	21,248	9,024	0

Temporal and aggregated degrees

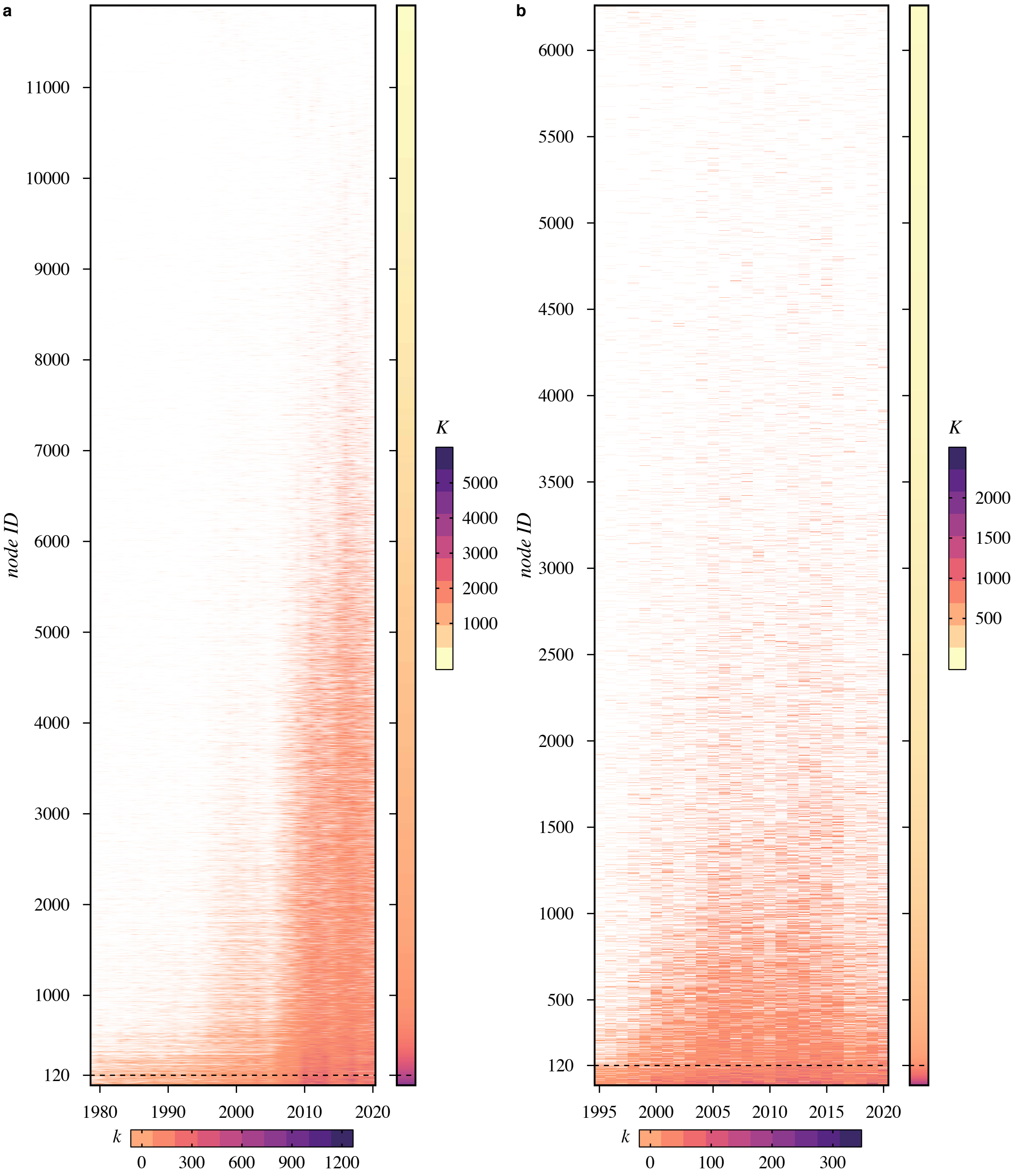

Figure S1: Raster plot with the node temporal degrees k and the aggregated degree K for (a) GDELT and (b) ICEWS. Node ids are descending order by the aggregated degree. The black dashed lines split the 120 hubs (below), which correspond to the 99th and 98th percentiles of the highest temporal degrees (K) for GDELT and ICEWS, respectively.
